# Putting the I in AML: Artificial Intelligence and Machine Learning in Acute Myeloid Leukemia

**DOI:** 10.3390/cells15171539

**Published:** 2026-08-26

**Authors:** Federico De Marchi, Giulia Ciotti, Alessandro Atanasio, Giovanni Pascarella, Alessandra Sperotto, Michele Gottardi

**Affiliations:** 1 Hematology Unit, Istituto Oncologico Veneto (IOV)—IRCCS, 35128 Padova, Italy; giulia.ciotti@iov.veneto.it (G.C.); michele.gottardi@iov.veneto.it (M.G.); 2 Laboratory for Transcriptome Technology, RIKEN Center for Integrative Medical Sciences, Yokohama 230-0045, Japan; giovanni.pascarella@riken.jp

**Keywords:** acute myeloid leukemia, artificial intelligence, machine learning, clinical readiness, measurable residual disease, precision medicine

## Abstract

Artificial intelligence (AI) and machine learning (ML) now reach into every stage of AML care. Deep learning models read therapy-relevant mutations directly from bone marrow smears; automated flow cytometry gating reproduces expert calls in under a minute; and the first AI pathology devices for hematology have cleared regulatory review and entered clinical use. Beyond diagnosis, ML captures the age-dependent weight of individual mutations that categorical ELN scoring misses, drug response prediction for venetoclax–azacitidine has been validated across multiple external cohorts, and large language models are being tested for tumor board support and trial matching. The next wave, from clonal architecture modeling and single-cell foundation models to digital twins and reinforcement learning for adaptive dosing, could move AML management from reactive toward predictive, evolution-aware care. This review departs from existing AI-in-hematology surveys in three ways: we (i) restrict the scope to AML and organize the field around clinical decision points rather than technology categories, (ii) grade every tool on a five-tier author-defined clinical readiness level (CRL-AML 1–5), which exposes hundreds of models clustered at CRL-AML 1–2 and none yet in prospective clinical evaluation, and (iii) close with a numbered three-year agenda naming the consortia, datasets, and pragmatic trials needed to carry the field from publication to practice.

## 1. Introduction

Acute myeloid leukemia is a family of molecularly distinct entities that share clonal expansion of myeloid precursors in the marrow but diverge in biology, prognosis, and response to treatment [1]. Reaching a diagnosis now means reconciling several layers of evidence at once. The 2022 WHO and International Consensus Classification (ICC) schemes, together with the ELN 2022 risk stratification and its 2024 update for less-intensive therapy, ask the clinician to fold morphology, immunophenotype, cytogenetics, and a widening panel of molecular markers into a single coherent assessment [1,2,3]. The quantitative burden is real: a typical AML genome carries roughly five recurrently mutated driver genes [4], on top of karyotypic abnormalities and serial measurable residual disease (MRD) readouts from flow cytometry and molecular assays. All of it must then be weighed against age, fitness, and comorbidity to settle on a treatment plan for one patient.

Even with targeted agents and venetoclax-based combinations, the five-year overall survival (OS) in AML still sits near 32%, reaching 40–50% only in younger, fit adults who achieve complete remission. Older patients fare worse, although VIALE-A established venetoclax–azacitidine as a standard for those unfit for intensive therapy, extending the median OS to 14.7 months against 9.6 months with azacitidine alone [1,5]. AI and ML offer one route through this complexity and possibly toward better cure rates. Over five years the field has matured from proof-of-concept studies to multi-center validations and, in a handful of cases, regulatory authorizations and openly available clinical tools [6,7,8].

A 2025 bibliometric analysis counted 376 original AI research articles in hematology, with AML being among the most studied diseases [9]. The work now ranges from deep learning that classifies bone marrow cells and infers mutations from slide images, through ML that resolves ELN risk into continuous estimates, to multi-cohort-validated drug response models for venetoclax-based regimens and large language model (LLM) tools for tumor board support and trial matching [6,7,8,10].

Volume, however, is not the same as clinical readiness, and the gap between a published model and a usable tool stays wide. Of 1016 AI/ML medical devices authorized by the U.S. Food and Drug Administration (FDA) through late 2024, hematology accounts for about 1.9%, some nineteen devices in all [11]. Prospective validation in AML is, for practical purposes, absent. Most models have been trained and tested at a few Western academic centers, which leaves open real questions of generalizability and equity [10].

Recent reviews have surveyed AI across hematology [6], focused on AML and myelodysplastic syndromes (MDS) [7], or systematically analyzed 426 publications from 2010 to 2024 [8]. The present review differs in three respects: its scope is restricted to AML; evidence is organized around clinical decision points rather than technology categories; and each tool is assigned an author-defined CRL-AML grade before a numbered three-year agenda identifies priority datasets, trials, and consortia.

For consistency, one author-defined clinical readiness level scale (CRL-AML) is applied throughout the tables:

**CRL-AML 1, Discovery:** Single-center, internal cross-validation only. CRL-AML 2, **Internal validation:** Held-out test set within the source cohort. **CRL-AML 3, External validation:** Independent cohort(s) from different institutions. **CRL-AML 4, Prospective evaluation:** Real-time clinical use studied prospectively, regardless of randomization. **CRL-AML 5, Regulatory authorization/clearance:** FDA, EMA, or equivalent authorization for clinical use. The CRL-AML scale is an explicit adaptation of the NASA Technology Readiness Level concept and of the FDA Total Product Lifecycle framework for Software as a Medical Device, recast to grade clinical rather than purely technological maturity; it is an organizing heuristic, has not been formally validated, and is not an established clinical or regulatory assessment standard (Section 8.3).

The review follows the clinical trajectory from suspicion and diagnosis through risk stratification, treatment, response monitoring, and transplantation. Each section considers the potential contribution, the strength of supporting evidence, and the barriers separating model development from clinical use. The final sections address foundation models, digital twins, and reinforcement learning in relation to the same translational criteria.

Throughout this review, ML denotes the subset of AI methods that learn from data; deep learning denotes multilayer neural networks, including convolutional networks for images and transformers for sequence and language; and large language models denote transformer-based systems trained on text. Much of the clinical literature still uses “AI” and “ML” interchangeably.

**Search strategy and scope.** PubMed, bioRxiv, and Crossref were searched for English-language records published between January 2015 and April 2026, pairing disease terms (“acute myeloid leukemia,” “AML,” “myelodysplastic syndromes,” “MDS-AML,” and “acute promyelocytic leukemia”) with method terms (“artificial intelligence,” “machine learning,” “deep learning,” “convolutional neural network,” “transformer,” “large language model,” “foundation model,” “graph neural network,” “reinforcement learning,” “federated learning,” and “digital twin”). Reference lists of reviews [6,7,8], the FDA AI/ML medical-device taxonomy [11], and the 2025 ELN-DAVID MRD consensus were hand-screened. Eligible records included primary research, methodological studies using AML or MDS-AML data, regulatory filings, and AML-relevant consensus statements; pure computer-science conference papers without clinical application, non-AML disease foci except when directly transferable, and editorials were excluded. Study selection was investigator-driven and prioritized reports addressing a defined AML decision point, an evaluable outcome, or a material validation or implementation issue. Screening was not exhaustive, no protocol was registered, no dual independent screening or formal risk-of-bias assessment was performed, and no PRISMA flow diagram or quantitative pooling was undertaken. The work is therefore a structured narrative review rather than a systematic review. Evidence is current as of mid-April 2026.

## 2. AI in AML Diagnosis

Diagnosis is where AI in AML has advanced furthest. Here the first regulatory clearances have arrived, the training datasets are largest, and the path into routine practice is shortest. Three modalities dominate the work: digital morphology, flow cytometry, and molecular classification.

### 2.1. Digital Morphology and Automated Blast Detection

Reading a bone marrow aspirate is among the oldest tasks in hematology and among the most subjective. Agreement between observers on manual blast counts is moderate at best, with κ values near 0.5 in published comparisons [12]. The consequences are not cosmetic: under ICC 2022 the 10% and 20% blast thresholds separate MDS, MDS/AML, and AML, so a disagreement near a cutoff can change both the diagnosis and the treatment. The WHO 5th edition drops the 20% threshold for AML defined by genetic abnormalities (*NPM1*, *KMT2A*-r, core-binding-factor fusions), yet the cutoff still governs the remaining categories. Morphology therefore became an early target for automation.

Convolutional neural networks handle blast detection well, and the evidence is now substantial. A systematic review and meta-analysis of AI for AML detection from peripheral blood images reported pooled accuracy above 0.95 [13], and broader surveys of laboratory hematology and hematopathology document similar gains in cell classification, malignancy detection, and integration with the full MICM (morphology, immunophenotype, cytogenetics, molecular) workup [14,15,16,17]. The more interesting models look past detection to genotype. Eckardt and colleagues trained a fully automated smear pipeline on 1251 AML patients and 236 healthy controls, reaching AUC 0.97 for AML versus normal and AUC 0.92 (95% CI 0.88–0.96) for *NPM1* status inferred from morphology alone, a same-day triage signal that arrives before confirmatory sequencing [18,19]. A later study pushed further with annotation-free multiple-instance learning on whole-slide images, reaching AUCs of 0.90 ± 0.08 for *NPM1* and 0.80 ± 0.10 for *FLT3*-ITD on a held-out set of 116 slides drawn from 572 cases [20]. With prospective validation, such tools could return preliminary molecular information within hours of biopsy; they cannot, however, replace the molecular workup that AML definition and later MRD monitoring require.

Generative modeling adds a complementary capability, exemplified by CytoDiffusion, a diffusion model trained on more than 500,000 peripheral blood smear images that classifies cells more accurately than trained hematologists and produces synthetic images that experienced observers could not tell from real ones in a blinded Turing test [21]. The same group released the largest public blood smear dataset to date, now available for benchmarking and model development.

Regulatory approval has arrived in two steps. In April 2024, Scopio Labs received the first De Novo FDA authorization for an all-digital bone marrow aspirate platform, the X100 Full-Field Bone Marrow Aspirate Application (DEN230034), creating a regulatory category for hematology AI where none had existed [22]. A 510(k) clearance in July 2025 extended it to red cell morphology across 23 parameters and platelet assessment, and CellaVision holds clearances for AI-assisted peripheral blood analysis in major markets [22]. Narrow as they are, these authorizations prove that a pathway exists, even if hematology remains a small minority within the broader FDA AI/ML device landscape [11].

One caveat runs under all of these results, namely that most were built on curated single-institution data, with standardized staining and clean imaging. How they behave in a routine laboratory, with different stain batches, older microscopes, and uneven slide preparation, is largely untested. External validation outside European adult cohorts is rare, and pediatric AML is almost absent. The AUCs reported on research cohorts therefore very likely overstate what these tools will achieve on unselected clinical material.

Of the diagnostic modalities, digital morphology is closest to routine use. Two vendors have cleared the regulatory path, and the input, a stained slide, is something every laboratory already produces. Whether mutation prediction from images actually shortens time to treatment will depend on how pathology workflows absorb these tools; for now that is a plausible promise rather than a demonstrated one.

### 2.2. AI in Flow Cytometry

Multiparametric flow cytometry (MFC) sits at the center of AML diagnosis and MRD assessment, and it is also among the most operator-dependent assays in the laboratory. Manual gating, the work of carving out cell populations in high-dimensional space, demands extensive training, takes 15 min or more per case, and gives results that drift between operators, instruments, and centers [23]. The European LeukemiaNet has issued consensus recommendations to standardize MFC-based MRD assessment [24,25].

Even so, residual variability still matters clinically: it limits the comparison of MRD data across trials and constrains the use of flow-based endpoints in regulatory submissions.

AI for flow cytometry divides into two tasks: diagnostic classification and MRD assessment. For classification, Cheng and colleagues trained a deep learning model on acute leukemia orientation-tube data, reaching 94.6% sensitivity for AML and 98.2% for B-ALL [26], and a more recent cross-institutional framework classified AML across heterogeneous panel designs at 98.15% accuracy and AUC 0.998 [27]. That second result matters because protocol variability between centers is one of the main obstacles to generalizable flow cytometry AI, and cross-protocol performance meets it directly. Lewis and colleagues went a step further, showing that a model trained on flow data can both diagnose AML and predict its molecular subtype, a fast proxy for genomic characterization [28].

For MRD quantification, the CCADDAS pipeline, built for B-ALL, cut analysis time from about 15 min to 1 min per case while reaching 100% positive agreement at thresholds of 0.01% or higher [29]. Its AML validation is still pending, since residual AML is immunophenotypically more varied, but the gains in speed and consistency carry over directly. A parallel from CLL makes a broader point: applied to multiparametric flow data, the ALPODS algorithm defined immune cell populations that predicted outcome better (AUC 0.95) than the standard CLL-IPI (AUC 0.78) [30], extracting signal that conventional gating leaves untouched.

No AI flow cytometry tool has yet earned FDA authorization, and AML explains much of the delay: its immunophenotype shifts between diagnosis and relapse, its blasts mimic regenerating normal progenitors, and residual cells near MRD-negative thresholds are vanishingly rare. The clinical incentive is nonetheless real because many community laboratories cannot perform high-quality flow MRD, and a validated automated pipeline would carry that capability beyond the academic centers that now hold it.

### 2.3. Genomic and Molecular Classification

Molecular classification poses a coordination problem of its own. Under both WHO and ICC 2022, assigning an AML diagnosis means integrating morphologic, immunophenotypic, cytogenetic, and molecular data through a multistep process that spans hematopathology, cytogenetics, and molecular laboratories [1,2].

Several groups have asked whether ML can shoulder this integration. The Unsupervised AML Multi-Omics Classification System (UAMOCS) combines genomic, methylation, and transcriptomic data into three subtypes whose prognostic profiles track the ELN 2022 scheme [31]. Others have used specific mutations and variant allele frequencies to refine the ELN 2022 categories themselves, recalibrating the existing system rather than supplanting it [32].

The most clinically suggestive results come from cheap, universally available inputs. An international validation of an AI algorithm predicting acute leukemia subtype from the complete blood count and basic biochemistry reached AUC 0.94 for AML, 0.98 for acute promyelocytic leukemia (APL), and 0.84 for ALL across 6206 patients in 20 centers [33]. These figures, however, hold only at a confidence cutoff that left 71–93% of cases unclassified; without it the real-world accuracy was lower (AUROC 0.84 for AML). Should it hold across varied settings, such a model could act as a rapid triage step, flagging a probable subtype before specialized testing returns. APL is the sharpest case, where any delay carries immediate mortality risk, and graph neural networks have been explored there for fast subtype identification [34].

Anticipating transformation to AML is a related and unsolved problem. In chronic myelomonocytic leukemia, a machine learning study combined hierarchical clustering with cooperative-mutation analysis to find co-mutation patterns that predict blast transformation, among them *NPM1*, *NRAS* + *SETBP1*, and *ASXL1* + *RUNX1*, and validated them in an independent Italian cohort [35]; conventional scoring had missed these interactions. Sequencing has meanwhile widened the molecular target itself, adding fusion transcripts, splicing variants, and non-coding RNA alterations to the features available for ML-based classification [36].

A different route to rapid classification comes from long-read sequencing itself. Because Oxford Nanopore devices call base modifications directly and stream reads in real time, a methylation profile builds during the run, and a classifier trained on array-based atlases can read it as it accrues. The approach was proven in neuro-oncology, where the Sturgeon network classified central-nervous-system tumors intraoperatively from sparse nanopore data in about 40 min [37], and unified assays such as ROBIN now add copy number, single-nucleotide variants, and fusions in one same-day workflow [38]. Acute leukemia has followed: MARLIN classified AML, B-ALL, and T-ALL from low-pass nanopore data against a 2540-sample, 38-class reference, returning subtype calls on prospective cases within about two hours [39], while the Acute Leukemia Methylome Atlas (ALMA) added a 38-CpG model predicting a five-year survival in independent pediatric and adult cohorts [40]. Methylation profiling thus offers what morphology and flow cannot, a single assay that assigns subtype and estimates outcome fast enough to inform the first treatment decision. The limits are real: low-pass nanopore gives sparse, largely binary methylation, and rare karyotypes stay undersampled in the training atlases; both classifiers remain externally validated but not yet prospective (CRL-AML 3).

None of these tools have reached the clinic, and AML raises a specific concern. The move from WHO 2016 to WHO/ICC 2022 already reclassified a meaningful share of patients, so layering an AI estimate on top adds a source of variability that current guidelines do not address. The upside is equally concrete: where the full WHO/ICC workup cannot be assembled quickly, in community hospitals, resource-limited settings, or emergencies that force an early decision, an AI approximation from routine data could remove days from the time to first treatment. Table 1 summarizes representative AI/ML tools across these diagnostic modalities.

## 3. AI in AML Risk Stratification and Prognosis

### 3.1. Machine Learning Models Extending the ELN 2024 Framework

The ELN 2022 stratification, updated in 2024 for patients on less-intensive therapy, sorts AML into favorable, intermediate, and adverse groups from cytogenetics and a lengthening list of molecular markers [2,3]. The scheme is useful but coarse. Two patients in the same group can follow very different courses, and three fixed bins capture neither the interactions between mutations nor the fact that risk is continuous.

The first thoroughly validated alternative was the knowledge bank model of Gerstung and colleagues, who joined multistage Cox modeling to hierarchical Bayesian shrinkage and integrated mutations, cytogenetics, age, and clinical features across 1540 patients [41]. Its individualized survival estimates outperformed the categorical ELN groups and have anchored the field ever since. Eckardt and colleagues then made the case for context: training age-stratified models on 3062 patients, they showed that a mutation’s prognostic weight shifts markedly with age [42]. A lesion that signals good prognosis in a young adult may be neutral, or adverse, in an older one. Categorical systems cannot express this because they assign each mutation a single fixed value regardless of the patient who carries it.

Later work has extended this logic to the patients ELN serves the least well. Beat-AML 2024 examined 595 adults aged 60 or older on lower-intensity therapy, where *IDH2* emerged as an independent favorable factor and *KRAS*, *MLL2*, and *TP53* as adverse; a combined “Beat-AML mutation score” defined groups with a two-year survival of 48%, 33%, and 11% [43]. The DATAML registry applied multilayer perceptrons to 3687 patients, reporting time-dependent accuracy of 68.5% for survival under intensive chemotherapy and 62.1% under azacitidine [44], figures that are clinically useful yet a reminder of how much individual-level uncertainty remains even with large models on large real-world data. A supervised model trained on 1383 patients predicted complete remission at AUC 0.77–0.86, with *U2AF1*, *IKZF1*, and bZIP-*CEBPA* carrying weight, and held at AUC 0.71–0.80 on a further 664 patients [45].

Machine learning clustering of older AML went further still, resolving nine genomic subtypes whose survival depended on whether treatment was intensive or not [46]. The implication is that optimal stratification may have to be treatment-specific rather than universal, a conclusion guidelines have yet to absorb.

A consistent picture emerges across these studies: ML beats categorical ELN stratification, modestly but reliably, with discrimination usually in the 0.7–0.8 AUC or C-index range and at least matching ELN 2022 within each study’s own validation cohort [41,42,43,44,45]. Two caveats temper this optimism, however: none of these models has been tested prospectively, and most were trained on intensively treated patients, whereas the growing venetoclax–azacitidine population, older and biologically distinct, is only beginning to enter the training data [47,48]. For now the models supplement existing risk stratification rather than replace it.

### 3.2. Transcriptomic and Multi-Omic Approaches

Gene expression carries prognostic information that mutation analysis only partly captures. The LSC17 score, a 17-gene signature that approximates leukemia stem cell burden, was among the first transcriptomic predictors developed and externally validated for AML, and it has held up across multiple cohorts [49]. Where mutation-based models read the genetic lesion, LSC17 reads the leukemia’s transcriptional state, and that distinction is clinically relevant: patients with identical mutations can diverge sharply at the expression level, and the divergence predicts outcome.

Newer work builds outward from expression alone in several directions. Silva and colleagues trained RNA-seq models on k-mer features, short nucleotide subsequences, exceeding 90% accuracy in risk prediction [50]; integrative analyses that fuse expression with DNA methylation through network-based feature selection improve on single-omic models [51]; and a multi-center study of epigenetic subtypes resolved two molecularly distinct groups with C-indices of 0.72–0.78 across validation cohorts [52].

Integration now reaches proteomics and metabolomics as well [53,54]. A 2025 spatial proteomic study profiled AML marrow by high-plex immunofluorescence and mass cytometry imaging and found tertiary lymphoid-like aggregates whose signatures were prognostic in an independent 480-patient transcriptomic cohort [55]. Methodologically, graph neural networks and tensor factorization are beginning to connect single-cell data to clinical prediction, handling modality prediction, matching, and joint embedding within one framework [53,54,56].

Risk stratification is moving from categorical groups toward continuous, individualized probabilities drawn from several data layers. What limits it now is not the algorithms but the assays. Multi-omic profiling needs RNA-seq, methylation arrays, proteomics, and increasingly metabolomics, none of which most clinical laboratories run routinely. The analytic methods exist; the infrastructure to generate the data at the point of care does not. NGS panels became routine once their cost fell far enough over the past decade, and whether multi-omic profiling follows the same curve will decide when these models reach the clinic.

## 4. AI in AML Treatment Decision Support

### 4.1. Drug Response Prediction

For most patients the first major treatment decision, between intensive chemotherapy, venetoclax–azacitidine, and a targeted agent, turns on age, fitness, and a few molecular markers. The approach is reasonable on present evidence but blunt: two patients sharing an ELN category and a frontline regimen can still diverge widely, and no dependable method predicts that divergence at the individual level.

The most advanced attempt is the RF8 model of Jin and colleagues for predicting response to venetoclax–azacitidine (VEN/AZA) [57]. Drawing on ex vivo drug sensitivity testing, transcriptomic profiling, and CRISPR screening, they distilled eight genes whose expression tracks VEN/AZA response and then built a random forest validated across four independent cohorts totaling 498 patients, with scores rising near-monotonically with both response probability and survival. Few AML treatment prediction models have been externally validated in several datasets, which makes RF8 the most plausible near-term candidate for prospective testing. Three limitations keep it short of the clinic: it rests on only eight genes, it needs RNA-seq input that not every center can provide, and all four validation cohorts were retrospective.

Other approaches widen the lens by representing the links between molecular features and drug sensitivity as networks. These knowledge graph models flagged expression signatures, among them *FGD4*-*MIR4519*, *NPC2*-*GATA2*, and *BCL2*-*NFKB2*, that predict ex vivo venetoclax response [58]. NetAML scaled the idea up, building 87 sensitivity models across 87 compounds in primary samples from 520 patients to yield a pan-drug atlas for AML [59]. Even mutation patterns alone, without expression, can predict sensitivity in some settings [60].

These predictors do not stand apart from biology; they increasingly fold it in. Monocytic differentiation, marked by loss of *BCL2* and a shift to *MCL1*-dependent survival, is enriched among patients with primary venetoclax resistance, though response is not uniformly lost in this group [61], and post hoc analyses of VIALE-A show that *TP53* and *RAS*-pathway co-mutations independently shorten responses to venetoclax–azacitidine [5]. ML models are beginning to incorporate such readouts rather than compete with them [52,55,57]. What is still missing is comparison: head-to-head evaluations of VEN/AZA predictors are scarce, and no prospective study has randomized patients to ML-based selection against standard criteria. The logical next step is a pragmatic trial that embeds RF8, or an equally validated predictor, as real-time decision support.

The question hematologists face most often, intensive 7 + 3 versus venetoclax–azacitidine, has itself been addressed by ML decision support tools, though these remain exploratory and untested prospectively [62]. At the far end of the treatment arc, MM-AI-AML couples mechanistic modeling of myelopoiesis with AI to predict the severity of chemotherapy-induced myelosuppression before treatment starts, at AUC 0.85 internally and 0.78 externally [63]. Toxicity prediction tends to be neglected next to efficacy, yet for older or frail patients, avoiding severe myelosuppression can matter as much as reaching remission.

Graph neural networks open a different line of inquiry, exemplified by CGMega, an explainable graph-attention network that organized 396 candidate AML genes into functional modules, yielding both predictions and mechanistic hypotheses about drug targets [64]. PDGrapher, a causally inspired framework, predicts combinatorial perturbations, sets of targets able to reverse a disease transcriptional state, with applications in drug repurposing and combination design [65,66].

None of these tools are in clinical use. Ex vivo sensitivity is an unreliable guide to in vivo response, and even RF8 awaits prospective testing. The trials that would settle the matter are overdue.

### 4.2. Large Language Models and Generative AI

Large language models are being tested for a different kind of work, not pattern recognition in molecular data but the reasoning that surrounds a clinical decision: synthesizing the literature, preparing cases, and matching patients to trials.

Schmutz and colleagues put ChatGPT-4.0 to 20 real-world molecular tumor board (MTB) cases across breast cancer, glioblastoma, colorectal cancer, and rare tumors [67]; the cohort was oncologic rather than AML-specific, but the lesson carries to hematologic boards. The model proposed more therapeutic options per case than the expert panel (median 3 vs. 1; *p* = 0.005) and needed far less preparation time (median 15.2 vs. 34.7 min; *p* < 0.001). It also exposed the characteristic weakness of current LLMs: the supporting evidence varied in quality, and some recommendations were poorly sourced, the price of a system that writes plausible and incorrect statements with equal fluency.

Trial matching shows the technology at its most useful. TrialGPT, built by the NIH National Library of Medicine and the National Cancer Institute, recalled more than 90% of relevant trials while screening under 6% of the candidate pool, with 87.3% accuracy at the criterion level and a 42.6% cut in screening time [68]. For AML centers running active trial portfolios, that speaks to a genuine bottleneck: under-enrollment that stems from the sheer difficulty of matching complex patients to the right protocol quickly.

Head-to-head comparisons temper the enthusiasm: tested against one another and against expert judgment for clinical evidence summarization in hematologic malignancies, Claude, GPT-4, Gemini, and Llama show performance that varies by task and model, with no system uniformly ahead [69,70]. Broader scoping reviews in oncology reach the same verdict: the technology is advancing fast, but validated clinical deployments remain scarce [71,72,73].

The clinical boundary is clear: LLMs should not make treatment decisions. A model that offers an option without grasping a patient’s comorbidities, prior toxicities, or circumstances is generating suggestions, not judgments. Their value lies in throughput, since a busy academic board reviews dozens of cases a week and halving preparation time compounds quickly [67]. Governance is catching up: early frameworks call for mandatory human review of all AI-generated clinical content, audit trails for LLM-assisted recommendations, and clear disclosure whenever these tools touch the medical record [71,72,73]. Table 2 summarizes representative ML models for AML risk stratification and drug response prediction.

## 5. AI in AML Response Assessment and MRD

### 5.1. AI-Assisted Flow Cytometry MRD

Automated MRD detection is not new, and it sets out to make MFC-based MRD faster and more reproducible [74]. Support vector machines were applied to the problem as early as 2016 [75], and by 2018 a clinically validated algorithm matched expert performance in AML and MDS [76]. The more recent MAGIC-DR, an XGBoost pipeline, reached a cross-validation median AUC of 0.97 on its training set (98 diagnostic AML plus 30 MRD-negative samples) and agreed encouragingly with expert gating on a separate 25-sample test set, though that test set is too small to be conclusive [77]. CCADDAS, discussed in Section 2.2, has set the B-ALL benchmark, but its AML validation is not yet complete [29].

Mocking and colleagues, surveying computational immunophenotypic MRD in AML, named the core problem: too many algorithms and no standard [74]. Tools differ in framework, gating strategy, and positivity threshold, and no multi-center study has yet compared them on a common sample set. The parallel is with the early days of molecular MRD, where the absence of an assay standard delayed MRD’s entry into trial endpoints by years.

A cloud-based, software-agnostic pipeline could bring reference-quality MRD analysis to the many laboratories that lack the expertise today. In AML trials, where MRD is increasingly used as a surrogate endpoint for approval, standardized automated reading would also strengthen the regulatory case. The 2025 ELN-DAVID consensus made harmonization its priority, issuing 56 recommendations across technical standards and clinical interpretation, and automated gating is one obvious route to implementing them [25].

### 5.2. Molecular MRD and Clonal Tracking

Sequencing has added a second dimension to AML MRD. Where flow cytometry counts residual blasts by immunophenotype, error-corrected NGS detects persistent somatic mutations down to variant allele frequencies of 0.01–0.1%, depending on the platform (duplex sequencing, unique molecular identifiers). Jongen-Lavrencic and colleagues established that molecular MRD at complete remission independently predicts relapse and survival [78], and Morita and colleagues refined the reading, showing that which mutations persist, and how fast they clear, carries prognostic weight beyond a simple positive-or-negative call [79].

The interpretation of molecular MRD is subtler than it first appears because not all persistent mutations carry the same weight. Jongen-Lavrencic and colleagues showed that persistent *DNMT3A*, *TET2*, and *ASXL1* (DTA) mutations at remission mark age-related clonal hematopoiesis and do not predict relapse, whereas persistent non-DTA mutations remain meaningful [78]. Telling true leukemic persistence from background clonal hematopoiesis therefore hinges on which mutations persist, not merely on whether any do, and this is where computation enters. Models trained on mutational patterns reclassify AML past the old primary-versus-secondary divide, reading genomic signatures that betray the disease’s clonal origin [80].

A more ambitious line asks whether clonal architecture itself, the relative abundance, order, and subclonal structure of mutations rather than their mere presence, predicts outcome. Analyzing 2829 patients, Benard and colleagues found branched clonal evolution to carry a better prognosis than linear accumulation and tied specific subclones to specific signals: subclonal *TP53* abundance to white cell count and blast percentage, subclonal *NRAS* to ex vivo drug sensitivity, and subclonal *IDH1* to both, none of which binary mutation status can see [81]. The clinical weight of hierarchy runs deeper still. Shlush and colleagues traced relapse to pre-leukemic stem cells carrying founder mutations, locating not just who relapses but from which compartment [82], and Hourigan and colleagues showed that conditioning intensity before transplant interacts with genomic MRD to shape outcome, a reminder that monitoring data only mean something in their therapeutic context [83,84].

The computational toolkit for this reconstruction is well developed, beginning with Bayesian methods such as PyClone and its scalable successor PyClone-VI, which infer subclones from bulk variant allele frequencies, while SciClone, PhyloWGS, and newer ML approaches extend reconstruction to phylogeny across sites and timepoints [80,81,85]. Targeted single-cell DNA sequencing has directly mapped mutation co-occurrence and clonal evolution across myeloid malignancies [86], whereas GoT links genotype to transcriptome within the same cell [87]. These data provide single-cell ground truth for structures that bulk methods can only infer.

These tools matter most against the resistance patterns that modern therapy produces. Venetoclax-based regimens have made the point vividly: response is often clonally heterogeneous, as sensitive clones collapse under selection while pre-existing *TP53* or *RAS*/MAPK subclones, monocytic populations, and emergent *FLT3* kinase-domain mutations expand to drive relapse [5,61]. The natural role for AI here is to follow these trajectories forward rather than reconstruct them after relapse.

These components have not yet been integrated. No tool today takes serial molecular MRD across timepoints, joins it to clonal evolution modeling, and guides decisions in real time, even though the raw material is in hand. Many patients now have sequential NGS from diagnosis through transplant, flow-based MRD at several timepoints, and, in research cohorts, single-cell clonal maps, and the reconstruction methods are mature. These pieces have simply never been connected into one system that updates individualized risk as each new result arrives.

This creates a specific clinical dilemma. A patient in morphological remission with a rising *TP53* variant allele frequency and a new *NRAS* subclone presents a question that guidelines do not answer: is this the start of relapse from a resistant clone about to dominate or clonal hematopoiesis drifting without consequence? Today the answer comes from judgment and time, from waiting for the next count, the next marrow, the next flow timepoint. A tool that reads the patient’s full clonal history against treatment context could estimate the probability of imminent relapse and, crucially, name the subclone driving it because a *TP53*-driven relapse demands a different response from one driven by *FLT3*-ITD or *IDH2*. Building it is the operational goal behind the adaptive dosing framework in Section 7.6.

### 5.3. Single-Cell and Spatial Technologies

Single-cell RNA sequencing has mapped the hierarchy of AML clones, picked out rare leukemia stem cell populations, and resolved the marrow immune microenvironment cell by cell [88,89]. Profiling the same patients through chemotherapy reveals how subpopulations expand or contract under pressure [90], and the GoT approach ties genotype to transcriptional state within a single cell, joining clonal identity to cell behavior [87].

Spatial transcriptomics adds a geographic dimension. In marrow from patients on pembrolizumab and decitabine, single-cell spatial analysis found immune cells clustering near leukemic ones after immunotherapy [91], and spatial proteomics identified tertiary lymphoid structures whose signatures predicted outcome in 480 patients [55,92].

For now these technologies describe rather than decide. No clinical AI tool runs on single-cell or spatial data, and the computational cost rules out routine use. As the assays grow cheaper and foundation models mature (Section 7.6), it should become possible to track not only whether residual disease persists but where it sits in the niche and how the surrounding microenvironment is arranged. That resolution is a long-term ambition, not a near-term deliverable. Table 3 summarizes AI approaches for MRD assessment, clonal architecture, and single-cell or spatial monitoring.

## 6. AI in Transplant and Cellular Therapy for AML

### 6.1. Predicting Transplant Outcomes and Post-HCT Relapse

The transplant decision in AML balances relapse risk against treatment-related mortality, weighing disease biology, donor characteristics, fitness, and MRD status together. The established scores, the Hematopoietic Cell Transplantation–Comorbidity Index (HCT-CI), the Disease Risk Index (DRI), and the EBMT score, were built with conventional statistics and capture only part of that complexity.

Masurekar and colleagues compared random survival forests, elastic-net Cox regression, and standard Cox models in 2253 patients undergoing allogeneic hematopoietic cell transplantation (allo-HCT), with external validation in 252 patients who had uniform MRD assessment [93]. Age over 60, MRD positivity, and adapted ELN adverse risk were the strongest predictors of poor outcome, yet the gain over conventional models was modest, which the authors read as a sign that pre-transplant variables alone may have hit a predictive ceiling. The contrast with time-updated data is instructive. The DERGA algorithm, applied to 564 consecutive adult allo-HCT recipients (AML among several underlying diseases), reached 93.26% accuracy for survivorship status by combining pre- and post-transplant features [94], and in a pediatric cohort, naïve-Bayes models using peri-transplant data from 30 days before to 30 days after transplant outperformed baseline-only predictors [95], pointing toward dynamic inputs as the way forward.

Relapse remains the most common cause of transplant failure, and several groups have aimed AI at predicting it. Texture and morphology features extracted from myeloblast chromatin in post-HCT aspirates predicted relapse at AUC 0.71, evidence that image-based biomarkers carry a post-transplant signal [96]. A dedicated model for relapse after haploidentical HCT in AML has appeared [97], and ML has mapped relapse patterns in pediatric transplant recipients with high specificity, albeit on small samples [98,99]. The most instructive example comes from another disease: Hernández-Boluda and colleagues built a random survival forest for transplant in myelofibrosis and released it as a free web calculator [100]. The biology differs, but the delivery model, a peer-reviewed algorithm paired with a maintained public tool, is exactly the template AML transplant tools will need if they are to change practice.

The pattern is consistent: ML adds incremental value over traditional scores, but reliable individual-level prediction is beyond reach with pre-transplant data alone. The next gains will come from post-transplant dynamics, from chimerism kinetics, serial MRD, early GvHD signals, and immune reconstitution. Transplant programs already collect these data, but models trained to learn from them are needed.

### 6.2. GvHD Prediction

Graft-versus-host disease (GvHD) is the leading cause of non-relapse mortality after allo-HCT, yet risk assessment still rests on donor matching, conditioning, and prophylaxis, all fixed at transplant and blind to the biology of the graft–host interaction as it unfolds. ML is being applied at both ends: before transplant, from clinical and genetic features, and after, from dynamic clinical data.

The pre-transplant work spans clinical, genetic, and molecular features. An ensemble of CatBoost, LightGBM, random forests, and logistic regression predicted acute GvHD at AUC 0.71–0.80 in 270 pediatric recipients, combining 53 clinical factors with 104 candidate single-nucleotide polymorphisms [101]. Targeted transcriptome profiling of pre- and post-transplant marrow yielded a 92-gene signature for acute GvHD and a 20-gene signature for survival [102]. Longitudinal EHR data have also been evaluated: in a 324-patient study, vital sign trajectories predicted acute GvHD before clinical onset [103].

The more promising direction is longitudinal because GvHD is a process rather than a single event. A model fed daily post-transplant laboratory values, vital signs, medications, and clinical events simply knows more than one limited to the pre-transplant snapshot. The early results bear this out: models built on longitudinal EMR data outperform baseline-only approaches for both acute and chronic GvHD [104].

Still, no GvHD model has been validated prospectively, and none yet guides prophylaxis in a trial [104]. The move from static pre-transplant scoring to continuously updated risk is no longer a technical problem but now a matter of trial design.

### 6.3. CAR-T and Novel Cellular Therapies

CAR-T-cell therapy has transformed the treatment of B-cell malignancies but has gained little ground in AML for one overriding reason: the disease offers no equivalent of CD19 [105]. Its blasts share surface antigens with normal myeloid progenitors, so every candidate target carries an on-target, off-tumor cost. Targeting CD33 produces prolonged cytopenias, as gemtuzumab ozogamicin showed long ago; CD123 constructs cause broad myeloid aplasia; CLL-1 leaves hematopoietic recovery uncertain. In several clinical programs this toxicity has capped both dose and durability [105]. Computational target discovery offers a way through. Single-cell transcriptomic atlases of leukemic and normal marrow, mined with machine learning surfaceome classifiers, can rank antigen combinations that cover the leukemia while sparing normal progenitors and can begin to match a patient to the construct most likely to engage their disease [106].

Outcome prediction is further along in lymphoma than in AML. In 416 patients given axicabtagene ciloleucel for diffuse large B-cell lymphoma, Wang and colleagues predicted relapse within six months from seven routine variables: age and six laboratory values (LDH, CRP, ferritin, hematocrit, platelet count, prothrombin time) [107]. Toxicity prediction has drawn similar attention, again by borrowing across diseases. Because severe cytokine release syndrome and COVID-19 share an inflammatory signature, models pretrained on COVID-19 cytokine-storm data have been transferred to predict CRS in CAR-T recipients where dedicated training data are scarce [108]. The same toolkit reaches upstream of the infusion as well, where computational-experimental pipelines nominate selective and synergistic drug combinations for relapsed AML [57] that could debulk resistant subclones before or after cellular therapy.

As CAR-T platforms against CD33, CD123, and CLL-1 and dual antigens move through AML trials, the prediction frameworks built in lymphoma will have to be adapted rather than borrowed wholesale. Toxicity models are the priority because on-target, off-tumor myelosuppression is a distinct and serious hazard with no real counterpart in B-cell disease [109].

## 7. Challenges, Regulatory Landscape, and Future Directions

### 7.1. The Validation Gap

Most tools in this review have been validated only internally, trained and tested on data from one institution, often by cross-validation rather than a true external holdout. External validation on independent cohorts is rare, and prospective validation in AML is essentially absent.

Internal cross-validation can overestimate transportability because development and test data share demographic, treatment, and laboratory biases. In DATAML, a survival model trained in Toulouse and tested in Bordeaux yielded an external AUC of 0.55 for azacitidine-treated patients when the selected feature set was used [44]. This AML-specific result shows why external performance should be reported alongside internal metrics. RF8 [57] and the Gerstung knowledge bank [41] remain notable because they were evaluated in independent cohorts.

The trial record makes the imbalance concrete. Against thousands of published models, a ClinicalTrials.gov analysis of AI in oncology found that registered prospective AI trials in hematology number in the single digits [110,111]. The FDA demands prospective evidence for most therapeutic interventions, and a consensus is forming that AI tools shaping clinical decisions should meet a comparable bar. Workable routes exist, adaptive designs that allow continuous model updates without sacrificing rigor, digitized workflows for efficient data capture, and pragmatic trials embedded in routine care [111], but none have been adopted at scale in AML.

The imbalance is structural: the field produces more models than it validates [111]. Until external validation becomes the default expectation for publication rather than the exception, clinical adoption will remain limited.

### 7.2. The Regulatory Landscape

Within the FDA AI/ML device taxonomy (about 19 hematology devices out of 1016; Section 1) [11,112], radiology and cardiology dominate the cleared products, the specialties with mature imaging pipelines and large standardized datasets. Scopio Labs and CellaVision (Section 2.1) are the hematology exceptions, and beyond them, few others exist [11,113].

Hematology poses regulatory problems of its own, beginning with its inputs. Flow cytometry panels, mutational profiles, and expression signatures lack standardized formats, and where radiology has DICOM as a universal image standard, hematology has no equivalent infrastructure. The FDA’s Good Machine Learning Practice framework and Predetermined Change Control Plans apply in principle but remain untested here [112,114], and most of the research tools in this review have no regulatory pathway at all.

The field divides along a clear line between two kinds of tool. Those nearest to clinical utility, digital morphology and automated MRD gating, could plausibly win authorization within a few years. Those with the greatest potential impact, drug response prediction, clonal evolution tracking, and adaptive treatment optimization, act on data types and decisions for which no regulatory framework yet exists. Closing that gap requires agreement among hematologists, regulators, and developers on evidence thresholds, endpoints, human oversight, and post-deployment monitoring. A recent hematology laboratory analysis identifies dataset shift, automation bias, liability, and recurring validation and monitoring costs as implementation hazards [115]; because it is a narrative medicolegal analysis rather than empirical AML validation, it is cited here only for governance framing.

### 7.3. Explainability and Clinical Trust

Accuracy alone does not earn a model a place at the bedside because a risk score that cannot point to a reason invites skepticism however good its numbers. Explanations are therefore a condition of clinical use.

SHAP and LIME have become the standard way to decompose a prediction into feature contributions [116], but feature weight is not the same as biological sense. The more persuasive explanations point to something a hematologist already recognizes. In AML, the attention-based SCEMILA framework does exactly that, flagging the diagnostic cells an expert would, atypical hypergranular promyelocytes in *PML*::*RARA*, cup-like blasts in *NPM1*-mutated AML, and myelomonocytic cells in *CBFB*::*MYH11*, with 89% concordance between high-attention cells and expert-labeled features [117]. That is explanation that travels to the bedside because it links model output to morphology clinicians trust. More general solutions, combining several explainability techniques into a trust-centered framework, have also been proposed [118,119].

This distinction is not academic because a model that leans heavily on CD33 might be reading real biology or merely a staining artifact, and only a hematologist who knows the disease can tell which. The real test of an explanation is whether it survives that scrutiny.

Explainability becomes harder as the underlying models grow more complex. A random forest built on ten clinical features can be explained in terms a clinician recognizes, but a graph neural network over multi-omic data cannot, at least not with today’s tools. Greater confidence requires causal or mechanistic explanations that relate predictions to established AML biology.

### 7.4. Data Equity, Bias, and Ethics

Most AML models are trained on data from academic centers in the United States and Western Europe. Patients from low- and middle-income countries, minoritized populations within high-income countries, and pediatric populations remain underrepresented. This is clinically relevant: multi-omic analysis of 100 patients with genomically confirmed African ancestry and 323 comparison patients identified ancestry-associated differences in mutation profiles and prognostic effects, including divergent associations for *NPM1* and *NRAS* [120]. A model developed predominantly in European-ancestry adults may therefore transfer poorly to populations with different genetic backgrounds, exposures, and treatment patterns.

As a minimum equity standard, AML studies intended for clinical use should report discrimination and calibration by age and sex and, when consent, sample size, and data quality permit, by self-identified race or ethnicity and genetically inferred ancestry. Sensitivity, specificity, missingness, and confidence intervals should be shown for each subgroup, with prespecified procedures for recalibration [120].

Equity is not only a question of who appears in the training data; it returns at the point of deployment.

Some tools are designed to help settings that lack specialist expertise, yet in practice AI may benefit most the institutions already equipped to adopt it, the large academic centers with digital pathology, comprehensive molecular profiling, and bioinformatics support, widening the gap between specialized and community care rather than closing it. Federated learning (Section 7.5) is a partial remedy, not a complete one.

Ethical questions follow close behind, and several remain unresolved: patient disclosure, institutional governance, error reporting, and liability when AI-guided treatment fails. A recent governance framework for generative AI in oncology offers retrieval-augmented generation (RAG) and human-in-the-loop workflows as practical safeguards, though neither has been tested across diverse real-world settings [121]. In the European Union, the AI Act (Regulation (EU) 2024/1689) requires transparency and human oversight for AI used in clinical decisions, even as implementation timelines for medical applications have slipped and no hematology-specific guidance yet exists [122]. These conversations need to run alongside model development, not wait until after deployment.

### 7.5. Federated Learning and Multi-Institutional Infrastructure

Pooling data across institutions would address small cohorts, limited diversity, and single-center bias at a stroke. In practice, privacy regulation, institutional governance, and competition make centralized sharing hard to arrange.

Federated learning offers a way around this obstacle by inverting the usual flow. Each site trains the model on its own data and shares only the resulting model updates, never the raw patient records. Early leukemia work has paired federated learning with low-rank adaptation for communication-efficient classification [123], and explainable federated transformers combining vision transformers with ClinicalBERT have been proposed for joint leukemia classification and staging across decentralized devices [124], though both are engineering demonstrations rather than validated clinical tools. The proof of principle comes from imaging, where multi-institutional consortia have shown federated training matching centralized performance in pan-cancer studies; the equivalent hematology evidence is thinner but building within big-data consortia such as the HARMONY Alliance [125].

The practical hurdles remain substantial: homomorphic encryption can slow training roughly fifteenfold, and cross-border model updates raise unsettled questions under GDPR and its counterparts. Most fundamentally, federation distributes computation but does not harmonize data: differences in quality, labeling, and panel composition persist between sites and can degrade a model even when the federation itself is sound [126,127].

Even so, federation is the most realistic route to the large, diverse datasets AML models need. Centralized repositories face legal, logistical, and competitive barriers that have proven hard to overcome even within a single country. For AML in particular, where any one center sees limited numbers and where molecular heterogeneity demands large cohorts to capture rare subtypes, multi-institutional collaboration is not optional. Federated frameworks that tolerate heterogeneous data while preserving privacy are the practical compromise between what the algorithms require and what institutions can give.

### 7.6. Emerging Technologies

None of the three technologies below have produced a clinical tool for AML, but they are moving fast enough that practicing hematologists will meet them within a few years.

**Foundation models for single-cell genomics.** These models bring the idea behind large language models into cell biology. A network is first trained on tens of millions of single cells with no labels attached, so it picks up features that carry over to many tasks, and is then fine-tuned on the specific problem at hand. scGPT, CellFM, and Nicheformer were pretrained on 33, 100, and 110 million cells [128,129,130]. The hope is that one such model could be reused for AML subtype classification, drug response prediction, and clonal state annotation. That has been harder than expected. Used as they are, without fine-tuning on the task in front of them, these models still trail simpler tools built for the job on several benchmarks [131,132].

**Digital twins.** A patient-specific model that simulates disease progression and treatment response in silico, a “digital twin,” has been endorsed by the NCI as a future paradigm for precision oncology [133,134]. In AML it would fold genomic profile, clonal architecture at diagnosis, MRD kinetics, drug pharmacokinetics, and clinical course into one continuously updated prediction. Synthetic patient generation for trial enrichment has been demonstrated and could supply AI-generated control arms [135,136], but a fully dynamic, continuously learning patient model remains aspirational. Network approaches that jointly model clonal populations, the marrow microenvironment, and the immune system may provide the mathematical backbone, though no working AML prototype has yet been reported.

**Reinforcement learning (RL) for adaptive therapy.** Here a model learns by trial and error: it takes an action, sees what happens, and adjusts to do better over time, without being shown labeled examples. In treatment, the actions are choices about drug, dose, and timing, and the aim is lasting disease control, which suits a disease whose management has to change as the cancer evolves.

The clearest example is prostate cancer, where Gatenby and colleagues withdrew and reintroduced abiraterone on PSA thresholds instead of dosing to the continuous maximum tolerated dose [137,138]. This evolution-informed schedule delayed resistance in metastatic castration-resistant disease. The goal there is to prolong control, not to eradicate the cancer. The closest AML analog is therefore not curative-intent induction but the lower-intensity regimens used in older or unfit patients. Azacitidine–venetoclax is the obvious case, where durable control and quality of life matter more than cure. Deep RL dosing has been tested in simulation, recently with Bayesian methods that account for pharmacokinetic variability between patients [139]. RL coupled to population PK/PD models has been tried for propofol anesthesia, warfarin, and several anticancer drugs [140], but none of this has reached AML. The obstacle is not the absence of feedback but its granularity and the cost of exploring. Marrow is already sampled in the first cycles of that regimen, and circulating blasts offer a cheap early signal when present. Yet response at the marrow and MRD level remains intermittent. Prostate adaptive therapy can be switched on and off reversibly. A policy that explored intensification in AML could not, and the harm would be irreversible. A workable system would therefore learn offline, from routinely collected trial and practice data, and stay close to standard care, with that lower-intensity setting the natural place to begin.

Yet AML fits the adaptive therapy profile almost perfectly: measurable clonal heterogeneity at diagnosis, serial MRD that tracks the disease, clear decision points at induction, consolidation, maintenance, and transplant, and a widening menu of targeted agents to combine or sequence. Adaptive trials that update a model mid-study are technically feasible and already run elsewhere in oncology. Bringing them to AML treatment optimization is among the most underexplored opportunities in the field.

### 7.7. Consolidated CRL-AML 3+ Registry

Most of the tools in this review sit at the lower end of clinical readiness. Table 4 gathers the exceptions, every AML AI tool here that has reached external validation (CRL-AML 3) or higher, in contrast with the large number of CRL-AML 1–2 tools in Table 1, Table 2 and Table 3. The final column now adds operational requirements and reported turnaround where available; complete per-sample implementation costs were not reported consistently in the primary studies.

Of the 21 entries, three are FDA-cleared digital morphology platforms (CRL-AML 5), and none has been evaluated prospectively as real-time decision support in AML (CRL-AML 4). Routine data models can return results within minutes after inputs are available, whereas nanopore, genomic, or expression-dependent tools inherit upstream assay turnaround of hours to days. No CRL-AML 3+ study reported a complete implementation budget.

## 8. Conclusions and a Three-Year Research Agenda

**Figure 1:**
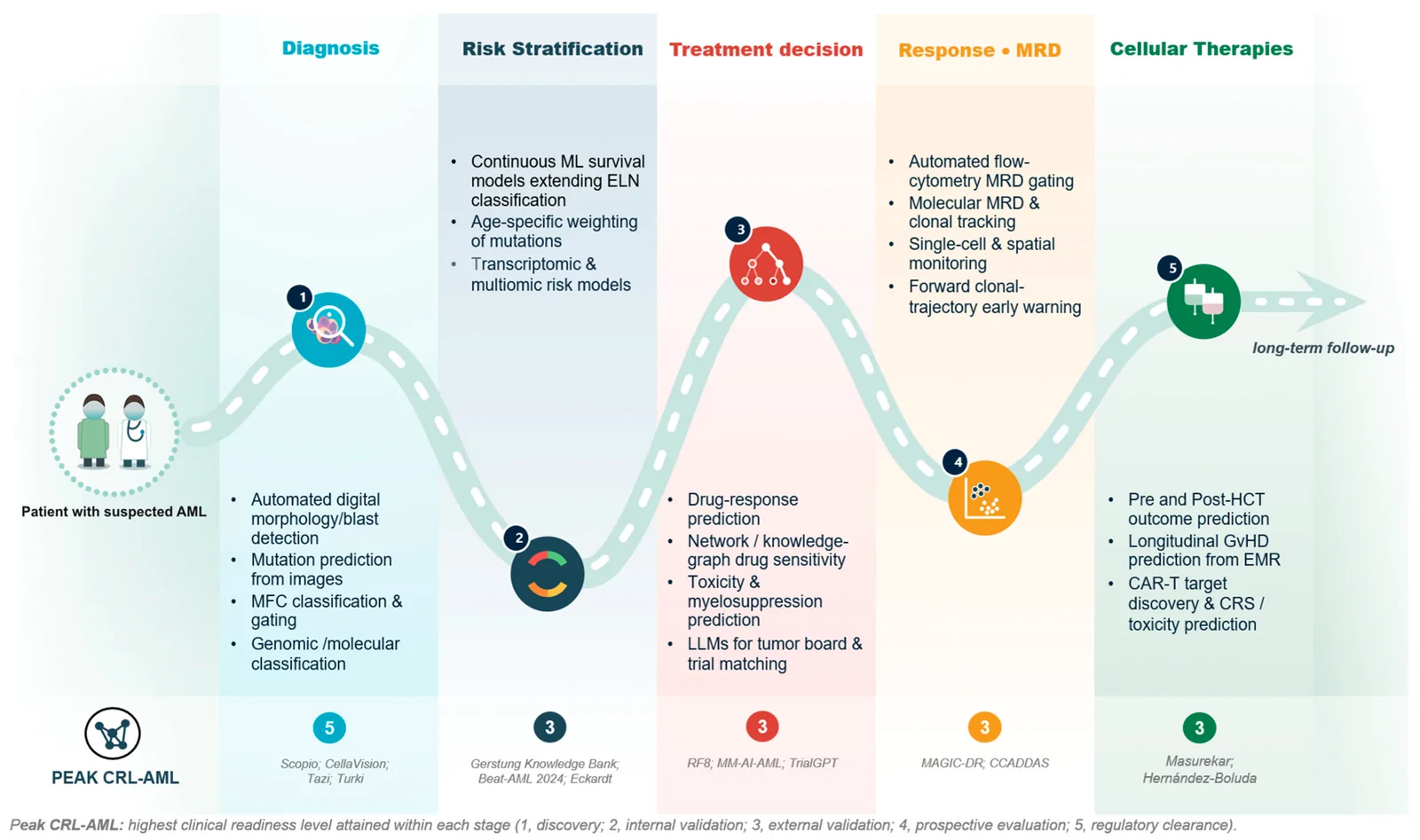
**The AML Patient Journey with AI and ML.** Overview of where AI and ML currently intervene along the AML care pathway, from suspected diagnosis through long-term follow-up. The five decision points are shown (diagnosis, risk stratification, treatment decision, response and MRD assessment, and cellular therapies), each annotated with the peak clinical readiness level reached by any tool in that domain (Peak CRL-AML) and with representative examples. The author-defined, non-validated readiness heuristic is: 1, discovery; 2, internal validation; 3, external validation; 4, prospective evaluation; 5, regulatory authorization. Only diagnostic tools have reached regulatory clearance; downstream applications have not progressed beyond external validation, and prospective clinical evaluation remains the principal translational gap. AML, acute myeloid leukemia; CAR-T, chimeric antigen receptor T cell; CRL, clinical readiness level; CRS, cytokine release syndrome; ELN, European LeukemiaNet; EMR, electronic medical record; GvHD, graft-versus-host disease; HCT, hematopoietic cell transplantation; LLM, large language model; MFC, multiparametric flow cytometry; MRD, measurable residual disease.

Figure 1 depicts the AML patient journey within a clinical framework in which AI and ML are integrated into clinical practice.

### 8.1. Where the Field Stands

Within this author-defined heuristic, no AI tool in AML has reached CRL-AML 4. Until one does, the claim that AI is “transforming AML care” describes a publication trajectory, not a clinical one.

By this scale, the overwhelming majority of tools reviewed here sit at CRL-AML 1–2, and only a handful, RF8, the Gerstung knowledge bank, the Turki international algorithm, and MAGIC-DR, reach CRL-AML 3. The FDA-cleared digital pathology entries, from Scopio and CellaVision, are graded CRL-AML 5 on regulatory status, but their predicate- and De Novo-based clearances came without a prospective, AML-specific utility trial, so regulatory status here does not amount to clinical validation. That gap, hundreds of CRL-AML 1–2 models against zero prospective evaluations, frames the next three years.

### 8.2. From Publication to Practice

The most useful next step is less about building new models than about testing the ones that already exist. Drug response prediction for venetoclax–azacitidine sits closest to a prospective trial, since its output maps onto a decision hematologists make: how intensively to treat. The clearest example is RF8 [57], so far the only externally validated response predictor of its kind (Section 4.1), though it rests on a single 2025 study and would need independent confirmation before it could anchor a trial. The broader point holds whichever model is used: a validated response predictor should be carried into a pragmatic randomized comparison with standard ELN 2024 care [3], with survival as the endpoint. That, rather than another retrospective model, is what would move the field toward its first prospectively validated tool.

The larger opportunity lies further out in adaptive, evolution-aware therapy. AML, with its measurable clonal heterogeneity and serial MRD, is well suited to treatment that is adjusted as the disease changes, yet by 2026 no AML-specific RL trial had been attempted. A sensible first step would be a small safety-and-feasibility study of model-guided dosing in consolidation, with stopping rules set in advance [135,136]. This would provide early evidence without allowing an algorithm to learn on patients in real time. Single-cell foundation models, by contrast, are still not ready for this: without task-specific tuning they still fall short of the supervised tools already in use [131,132], and they belong in the laboratory until benchmarks show otherwise.

None of this will matter without some shared groundwork. External validation in at least one independent cohort should become the minimum bar for any AML model that claims clinical relevance, much as CONSORT-AI and SPIRIT-AI did for trial reporting. Flow cytometry MRD is the natural place to begin: a shared, federated reference dataset, assembled across countries and harmonized under the 2025 ELN-DAVID consensus [25], would help with both standardization [24,74] and unequal access to data [141,142]. Regulators will need to keep pace, since without an agreed FDA–EMA view of what evidence suffices for AI in MRD, risk stratification, and drug response prediction [112,114], little beyond digital morphology will reach the clinic. The tool that would matter most to patients still does not exist: a model that reads a patient’s clonal trajectory and turns it into an individual relapse risk at the points where treatment is actually decided (Section 5.2). Building it and testing it prospectively should be the priority for the next few years.

### 8.3. Limitations of This Review

This is a structured narrative review, not a PRISMA systematic review or meta-analysis. Study selection was investigator-driven rather than exhaustive; no protocol was registered, no dual independent screening or formal risk-of-bias assessment was performed, and no quantitative pooling was undertaken. English-language sources were emphasized, leaving work published in other languages underrepresented. The field moves quickly, so each claim is a snapshot as of mid-April 2026. CRL-AML 1–5 is an author-defined organizing heuristic that has not been validated against external frameworks, regulatory decisions, or clinical outcomes and should not be interpreted as an established assessment standard. Table 5 makes the methodological and scope differences from references [6,7,8] explicit.

### 8.4. Coda

AML has what it takes to be the disease where AI in hematology earns a clinical role: genomic complexity, measurable clonal dynamics, well-defined decision points, and a strong cooperative-group infrastructure. The next three years will depend less on building new models than on running the trials that test the ones already on the table.

## Figures and Tables

**Table 1:** Representative AI/ML tools in AML diagnosis, by modality and evidence maturity. CRL-AML is an author-defined, non-validated heuristic: 5, regulatory authorization; 3, external validation; 2, held-out or partial internal validation; 1, internal cross-validation only. Compound metrics are labeled by task.

Modality	Tool/Reference	Task	Key Metric	Cohort	CRL-AML	Regulatory Status
Digital morphology	Eckardt 2022 [18]	AML detection; *NPM1* status prediction from bone marrow smears	AML detection: AUC 0.97 *NPM1* prediction: AUC 0.92	1251 AML + 236 controls (*NPM1* subset *n* = 408)	3	Research
Digital morphology	Kockwelp 2024 [19]	Therapy-relevant genetics from BM smears	—	Single-center	2	Research
Digital morphology	Wei 2025 [20]	*NPM1* status and *FLT3*-ITD prediction from whole-slide images	*NPM1* prediction: AUC 0.90 *FLT3*-ITD prediction: AUC 0.80	572 WSI single cohort	2	Research
Digital morphology	CytoDiffusion [21]	Generative blood cell classification	Turing-level vs. 10 experts	>500 k images	3	Research
Digital pathology	Scopio Labs FF-BMA [22]	Full-field BM aspirate digital analysis	—	—	5	FDA De Novo (Apr 2024) + 510(k) (Jul 2025)
Digital pathology	CellaVision [22]	Peripheral blood differential	—	—	5	FDA-cleared (multiple)
Flow cytometry	Cheng 2024 [26]	AML detection; B-ALL detection	AML sensitivity: 94.6% B-ALL sensitivity: 98.2%	Single-center	1	Research
Flow cytometry	Wang 2025 [27]	Cross-institute AML classification	98.15% acc, AUC 0.998	Multi-center, heterogeneous panels	3	Research
Flow cytometry	Lewis 2024 [28]	AML diagnosis + molecular subtypes	—	Single-center	1	Research
Molecular classification	UAMOCS [31]	Multi-omics AML subgroups, ELN-aligned	3 classes	Pooled datasets	2	Research
Molecular classification	Tazi 2022 [32]	Unified molecular classification + risk	16 classes covering 100% pts	3653 pts	3	Research
Molecular classification	Turki 2026 [33]	Acute leukemia subtype from routine labs	AML: AUC 0.94 APL: AUC 0.98 ALL: AUC 0.84	6206 pts, 20 centers	3	Research
Molecular classification	MARLIN [39]	Nanopore methylation subtype (AML/B-ALL/T-ALL)	Retrospective: 25/26 concordant Prospective: 5/5 concordant	2540 reference; prospective real-time	3	Research
Molecular classification	ALMA [40]	Methylation AML subtype + 5 yr survival	38-CpG survival model	3314 across 11 cohorts	3	Research

**Table 2:** Representative ML models for AML risk stratification and drug response prediction. CRL-AML is the author-defined, non-validated heuristic used in Table 1. n/a, not reported.

Model/Reference	Task	Features	Cohort	CRL-AML	vs. ELN/Standard	Deployment
Gerstung knowledge bank [41]	Individualized survival	Mutations + cytogenetics + clinical	*n* = 1540	3	Outperforms categorical ELN	Research tool
Eckardt age-stratified [42]	Age-specific molecular weighting	14+ mutations × age bins	*n* = 3062	3	Identifies divergent age effects	Research
Beat-AML 2024 [43]	ELN refinement for lower-intensity Tx	ELN + *IDH2*, *KRAS*, *MLL2*, *TP53*	Beat-AML cohort	3	Refines ELN 2024	Research
DATAML [44]	OS under IT vs. AZA	Clinical + mutations	*n* = 3687	1	Comparable to ELN	Research
Eckardt CR-supervised [45]	Complete remission prediction	Mutations + clinical	*n* = 1383	2	AUC 0.77–0.86	Research
Silva transcriptomic [50]	Risk from k-mer RNA features	RNA-seq	*n* = 404	2	>90% accuracy	Research
RF8 (Jin) [57]	VEN/AZA response	8-gene random forest	*n* = 498 pooled	3	Outperforms mutation-only	Closest to clinical-ready
Knowledge graphs (Qin) [58]	Drug response	Network + expression	—	2	n/a	Research
NetAML [59]	87-drug sensitivity atlas	Network + molecular	*n* = 520 ex vivo	2	n/a	Research
Qin mutation patterns [60]	Drug sensitivity from mutations	Mutational profile	Clinical cohort	2	n/a	Research
Islam 7 + 3 vs. VEN/AZA [62]	Treatment selection	Clinical + molecular	Retrospective	1	—	Exploratory
MM-AI-AML [63]	Myelosuppression severity	51 clinical features + TabNet	*n* = 479 + 900 virtual	3	No standard comparator	Research

**Table 3:** AI approaches for MRD assessment, clonal architecture, and single-cell or spatial AML monitoring. Compound metrics are labeled by task. n/a, not reported.

Tool/Reference	Assay	Task	Metric	Cohort	Status
Ni 2016 (SVM MRD) [75]	Flow	First automated AML MRD gating	Expert-level	Single-center	Historical
Ko 2018 [76]	Flow	Validated AML/MDS MRD	Concordance with experts	Multi-center	Validated
MAGIC-DR [77]	Flow	Interpretable AML MRD (XGBoost)	Internal cross-validation: median AUC 0.97 Separate test set: *n* = 25	Single-center	Research
CCADDAS (B-ALL) [29]	Flow	MRD pipeline	Manual analysis: approximately 15 min Automated analysis: approximately 1 min Agreement: 100% at 0.01% or higher	Multi-center	Research
Mocking review [74]	Flow	Computational MRD landscape & standardization gap	—	n/a	Review
Jongen-Lavrencic NEJM [78]	NGS	Molecular MRD at CR as relapse predictor	Overall survival: independent predictor Event-free survival: independent predictor	Multi-center	Reference standard
Morita mutation clearance [79]	NGS	Kinetics of somatic mutation clearance	—	—	Foundational
Awada ML genomics [80]	NGS	ML subclassification beyond primary/secondary AML	—	—	Research
Benard clonal architecture [81]	Bulk NGS	Clonal outcomes + drug sensitivity	Branching > linear survival	*n* = 2829	Research
Shlush [82]	NGS	Pre-leukemic HSC origins of relapse	—	—	Foundational
Miles single-cell mutation analysis [86]	scDNA	Single-cell clonal evolution in myeloid malignancies	—	123 patients; 146 samples	Research
Nam GoT [87]	scRNA + genotype	Clonal transcriptomes	—	Multiple MPN/AML cohorts	Research
Naldini longitudinal [90]	scRNA	Chemotherapy response dynamics	—	AML pts	Research
Gui spatial [91]	Spatial	Niche remodeling on immunotherapy	—	AML on pembro + DAC	Research
Ly spatial proteomics [55]	Spatial	TLS signatures predictive of outcome	Validated in *n* = 480	AML pts	Research

**Table 4:** CRL-AML 3+ tools across the AML care continuum, using the author-defined, non-validated heuristic. The final column summarizes clinical access and operational burden where reported. TAT, turnaround time.

Tool/Reference	Modality/Stage	CRL-AML	External Validation Evidence	Clinical Access/Operational Note
Scopio Labs FF-BMA [22]	Digital morphology—bone marrow aspirate	5	—	FDA De Novo (DEN230034, Apr 2024); deployed
Scopio Labs RBC + platelet morphology [22]	Digital morphology—peripheral blood	5	—	FDA 510(k) (Jul 2025); deployed
CellaVision DM-/DC-series [22]	Digital morphology—peripheral blood differential	5	Multi-site clinical use over >15 years	FDA-cleared (multiple); EU CE-IVDR; widely deployed
Eckardt 2022 *NPM1*-from-BM-smear [18]	Digital morphology—mutation prediction	3	Independent test cohort within multi-center dataset	Research
Wang 2025 cross-institute flow [27]	Flow cytometry—AML diagnosis	3	Heterogeneous panels across institutions	Research
Tazi 2022 unified classification [32]	Genomic—molecular subtyping + risk	3	*n* = 3653 across multiple cohorts; open access decision support tool released	Research; web tool
Turki 2026 international AI [33]	Routine-lab triage—acute leukemia subtype	3	*n* = 6206 across 20 international centers	Research; routine CBC and biochemistry; computation under 1 min; no additional assay
MARLIN (Steinicke 2025) [39]	Diagnosis—nanopore methylation subtyping	3	25/26 retrospective concordance; 5/5 real-time prospective	Research; nanopore library preparation and sequencing; classification approximately 2 h; per-sample cost not reported
ALMA (Marchi 2025) [40]	Diagnosis/prognosis—methylation atlas	3	Independent pediatric + adult cohorts across 11 harmonized datasets	Research; methylation assay plus classifier; upstream TAT depends on platform; cost not reported
Gerstung knowledge bank [41]	Risk—individualized survival	3	Validation across multiple AML cohorts	Research; web calculator; uses existing clinical and genomic data; inherits upstream genomic-testing TAT
Eckardt age-stratified [42]	Risk—age × mutation interaction	3	*n* = 3062 pooled multi-cohort	Research
Beat-AML 2024 ELN-refined [43]	Risk—older adults on lower-intensity therapy	3	Held-out validation within Beat-AML	Research
Eckardt CR-supervised [45]	Risk—complete remission prediction	3	*n* = 664 external validation cohort	Research
RF8 (Jin 2025) [57]	Treatment—VEN/AZA response	3	*n* = 498 across four independent cohorts	Research; eight-gene expression input; inherits upstream RNA-assay TAT; study cost not reported
MM-AI-AML [63]	Toxicity—myelosuppression severity	3	Internal + external (AUC 0.78)	Research
MAGIC-DR [77]	Flow MRD—interpretable XGBoost	3	Concordance with expert gating	Research
CCADDAS [29]	Flow MRD—B-ALL (AML pending)	3	Multi-center, cloud-based pipeline	Research cloud pipeline; approximately 1 min versus 15 min manual analysis; local-validation cost not reported
Masurekar 2026 transplant [93]	Transplant—pre-HCT survival	3	*n* = 252 external cohort with uniform MRD	Research
Hernández-Boluda 2025 (myelofibrosis) [100]	Transplant—survival post-HCT	3	Random survival forest with public web tool	Research; web calculator
Schmutz ChatGPT-4 MTB [67]	LLM—molecular tumor board	3	Real-world MTB cases (oncology, not AML-specific)	Research; median preparation 15.2 min versus 34.7 min for experts; integration cost not reported
TrialGPT [68]	LLM—patient–trial matching	3	Validation across three cohorts; user study	NIH research system; screening time reduced by 42.6%; integration cost not reported

**Table 5:** Direct comparison of the present review with references [6,7,8]. CRL-AML, clinical readiness level for AML; MDS, myelodysplastic syndromes.

Review	Disease Scope	Evidence Base/Time Window	Organizing Principle	Readiness Grading	Agenda-Setting
Nazha et al. [6]	Hematology-wide	Selected literature current to 2025; number of studies not specified	Data modality and hematology application	No tool-level scale	Broad future directions
Al-Nusair et al. [7]	AML and MDS	Selected literature current to 2025; number of studies not specified	Diagnosis, prognosis, treatment prediction, and research tools	No tool-level scale	General translational requirements
Sabile et al. [8]	Acute leukemias	426 publications, 2010–2024	Systematic narrative synthesis of clinical problems and methods	No grade assigned to each tool	Recommendations for clinical actionability
Present review	AML only	PubMed, bioRxiv, and Crossref, January 2015–April 2026; reference list screening	Clinical decision points along the AML care pathway	CRL-AML 1–5, author-defined and non-validated	Numbered three-year research agenda

## Data Availability

No new data were created or analyzed in this study. Data sharing is not applicable to this article.
